# Bismuth-Nanoparticles-Embedded Porous Carbon Derived from Seed Husks as High-Performance for Anode Energy Electrode

**DOI:** 10.3390/ma16206628

**Published:** 2023-10-10

**Authors:** Wasif ur Rehman, Umar Farooq, Muhammad Zain Yousaf, Ali Altalbe

**Affiliations:** 1 Hubei Key Laboratory of Energy Storage and Power Battery, School of Mathematics, Physics and Opto-Electronic Engineering, Hubei University of Automotive Technology, Shiyan 442002, China; wasif@huat.edu.cn; 2 Key Laboratory of the Ministry of Education for Advanced Catalysis Materials, Zhejiang Normal University, Jinhua 321017, China; 3 School of Electrical and Information Engineering, Hubei University of Automotive Technology, Shiyan 442002, China; mzainy1@gmail.com; 4 Department of Computer Science, Prince Sattam Bin Abdulaziz University, Al-Kharj 11942, Saudi Arabia; a.altalbe@psau.edu.sa; 5 Faculty of Computing and Information Technology, King Abdulaziz University, Jeddah 21589, Saudi Arabia

**Keywords:** biomass-derived carbon, bismuth nanoparticles, volume expansion, anode, stable performance, electrode

## Abstract

In energy application technology, the anode part of the electrode is typically composed of carbon-coated materials that exhibit excellent electrochemical performance. The carbon-coated electrodes facilitate electrochemical reactions involving the fuel and the oxidant. Energy electrodes are used in stationary power plants to generate electricity for the grid. These large-scale installations are known as distributed generation systems and contribute to grid stability and reliability. Understanding the practical applications of energy materials remains a significant hurdle in the way of commercialization. An anode electrode has one key limitation, specifically with alloy-type candidates, as they tend to exhibit rapid capacity degradation during cycling due to volume expansion. Herein, biomass-derived carbon from sunflowers (seeds husks) via pyrolysis and then bismuth nanoparticles are treated with carbon via a simple wet-chemical method. The electrode Bi@C offers several structural advantages, such as high capacity, good cycling stability, and exceptional capability at the current rate of 500 mA g^−1^, delivering a capacity of 731.8 mAh g^−1^ for 200 cycles. The biomass-derived carbon coating protects the bismuth nanoparticles and contributes to enhanced electronic conductivity. Additionally, we anticipate the use of low-cost biomass with hybrid composition has the potential to foster environment-friendly practices in the development of next-generation advanced fuel cell technology.

## 1. Introduction

The need for efficient energy storage solutions is critical, spanning a wide range of applications such as electric vehicles, consumer electronics, and the utilization of intermittent and renewable energy sources. Fuel cells and lithium-ion rechargeable batteries are widely recognized as excellent systems for good storage of electric energy, which can store and convert chemical energy into electricity efficiently and vice versa [1,2,3]. Nonetheless, the current predominant commercial anode material, graphite (840 mAh cm^−3^), possesses a limited capacity that falls short of meeting future demands [4,5]. On the other hand, low lithiation/delithiation voltage compounds have potential safety issues [6]. Hence, there has been a significant drive to discover and engineer high-performance anode materials and therefore to explore other families to enhance the overall electrochemical performances, like bismuth (Bi) element tin and manganese [7,8,9]. As mentioned, these families have unique properties such as high theoretical capacities, appropriate energy storage potential with Li^+^/Li, and eco-friendly characteristics [10,11].

In recent times, bismuth (Bi) has emerged as a highly promising candidate for lithium-ion battery anode materials, primarily attributed to its exceptional volumetric capacity (3765 mAh cm^−3^) and its moderate operating voltage range (0.5~0.9 V vs. Li^+^/Li) [12,13]. Featuring a characteristic layered crystal structure, bismuth (Bi) offers ample room to host lithium within its face-centered cubic (FCC) lattice, encompassing both tetrahedral and octahedral voids [14]. There are some challenges with bismuth (Bi) electrodes as the anode is constrained in cycling durability, dramatic feeding, and volume expansion, which is a common challenge seen in other high-capacity alloy-type anode materials such as silicon (Si), tin (Sn), aluminum (Al), and antimony (Sb) [3,15,16]. The primary reason behind this instability lies in the low electrical conductivity and substantial volume fluctuations occurring during the charge and discharge processes [17,18]. Numerous successful approaches have been employed to overcome this challenge, with the widespread adoption of tactics such as incorporating carbon and creating unique structures like nanosheets, nanorods, and nanowires. Indeed, Bi-based materials, when thoughtfully designed in combination with various carbon additives, have proven effective in mitigating volume fluctuations, preventing particle agglomeration, and improving the electrical conductivity of the electrode [19,20]. Researchers are investigating using carbon materials to prevent volume expansion during the cycling process to address these challenges. There is no such study, to our knowledge, that derives carbon from biomass sources to compact coated bismuth nanoparticles for anode electrodes. Biomass-derived carbon is a promising material for various energy storage applications. It highly contributes to cycling stability, enhanced rate capability, and suitability. The abundance and cost-effectiveness of carbon derived from biomass position it as a prime candidate for tackling the aforementioned challenges in supercapacitors [21,22], lithium-ion batteries [23], and sodium-ion batteries [24]. Yin et al. engineered carbon nanofibers with one-dimensional structures incorporating bismuth (Bi/C nanofibers) for a better understanding. This network of nanofibers with carbon demonstrated a high capacity of 309 mAh g^−1^ when cycled at 100 mA g^−1^ for 50 cycles [25]. Similarly, remarkable specific capacity results have been obtained using nanostructured Bi@C, displaying a performance of 300 mAh g^−1^ even after 100 cycles at 100 mA g^−1^ [26]. However, the battery performance of the Bi@C composite has fallen short, primarily due to the clustering of nanoscale materials during electrochemical processes. Therefore, the intentional design of the material structure is crucial in improving the lithium storage capabilities of anode materials based on bismuth (Bi). It has been further investigated that reducing the dimensions of Bi-based materials and introducing carbon prove advantageous for expediting electrochemical kinetics reactions and bolstering the internal conductivity of electrodes [27]. On a broader scale, ensuring structural integrity becomes paramount in enhancing cyclic stability during charge/discharge processes. Consequently, there is a pressing need for innovative Bi-based materials characterized by carbon wrapping as anodes.

**Figure 1:**
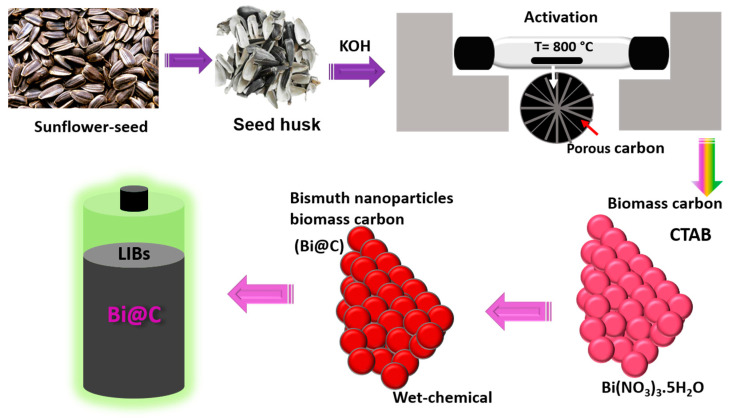
Scheme diagram of biomass-derived carbon from seed husks treated with Bi nanoparticles.

In this study, we present a simple two-step strategy for preparing Bi nanoparticles encased in a biomass-derived carbon coating, referred to as Bi@C nanocomposite. This procedure involves a two-step process: first, biomass-derived carbon from sunflower seeds is produced through pyrolysis at a temperature of 800 °C. The second step involves the incorporation of a bismuth precursor with carbon using a wet-chemical method and, finally, annealing the composite material under an argon flow. As illustrated in Figure 1, we employ lab-synthesized simple two-step Bi@C composite. The obtained free space configuration plays an important role and offers enough free paths to manage the volume change that accommodates the bismuth (Bi) during the electrochemical processes. Additionally, the ultrathin carbon coating remains non-adherent, ensuring enhanced stability. Furthermore, the carbon layer plays a crucial role in preventing the extraction or aggregation of Bi nanoparticles during repetitive charge/discharge cycles, thereby enhancing the structural stability of the Bi@C nanocomposite. More importantly, the carbon can boost Li^+^ ion and electronic conductivity, further expediting the kinetics reaction in the composite Bi@C electrode for the anode.

## 2. Experimental Section

### 2.1. Preparation of Biomass-Derived Carbon

Biomass carbon was obtained from sunflower seed husks, which were initially dried, ground physically to convert into a fine powder, and subsequently mixed with a 1:1 ratio of KOH. The mixture was subjected to calcination at 800 °C for one hour in a heating furnace. For more purification, the product was washed with HCl and deionized water several times to eliminate impurities. In the final stage, the product dried in an oven at 70 °C overnight. The obtained product was black in color to use for further experiments.

### 2.2. Preparation of Composite Bi@C

To obtain the desired composite of the Bi@C nanocomposite, a typical synthesis involved (Bi(NO_3_)_3_∙5H_2_O, 0.97 g) and CTAB 0.3 g mixed in DI water being added dropwise into 60 mL of diethylene for 1 h for strong sonication. Gradually, 0.1 g of the biomass-derived carbon was introduced; then the temperature was increased up to 110 °C, and this process continued for four hours in an ice bath. Afterward, the product was washed with deionized ethanol and water and then placed in a vacuum for 12 h at 80 °C. Ultimately, the collected sample was subjected to calcination at 550 °C for a duration of 2 h, with a gradual heating rate of 2 °C per minute, while maintaining an argon atmosphere to promote carbonization and facilitate the crystallinity of the Bi@C composite.

### 2.3. Structural Characterization

In order to scrutinize the microstructure and morphology of the Bi@C composite, we employed a diverse set of analytical techniques, including X-ray diffraction (XRD, Bruker, D8-Advance, Billerica, MA, USA). Using the testing instrument of (Horiba, LabRAM HR 800, Tokyo, Japan) to conduct Raman spectroscopy and also for physical characterization, field-emission scanning electron microscopy (SEM, JEOL, FEG250, Tokyo, Japan) Japan and transmission electron microscopy (TEM, JEM, F200, Tokyo, Japan) Japan was used. We also examined the states of surface chemicals with X-ray photoelectron spectroscopy (XPS) using a Thermo Fisher instrument (ESCALABXi_+_, Tokyo, Japan). To analyze the specific surface area of the product, we employed the Brunauer–Emmett–Teller (BET) method, utilizing a Micromeritics instrument (ASAP 2020 Plus HD88, Lafayette, LA, USA). Additionally, we performed thermogravimetric analysis (TGA) using a Mettler-Toledo instrument (TG-DSC, Geneva, Switzerland) under standard atmospheric conditions, employing a heating rate of 10 °C per minute with temperature from 25 °C to 700 °C.

### 2.4. Electrochemical Characterization

For the investigation of the electrochemical performance of Bi@C anodes, we prepared a homogeneous slurry comprising 80% active powder material, 5% acetylene black, and 15% polyvinylidene fluoride (PVDF). This slurry was subsequently applied onto a copper foil and dried under vacuum conditions at 90 °C for 24 h. Half coin cells of the 2025 type were meticulously assembled within an argon-filled glove box, with lithium foils serving as counter electrodes. The electrolyte solution was composed of 1 M LiPF6 dissolved in a mixture of dimethyl carbonate (DMC), ethylene carbonate (EC), and diethyl carbonate (DEC) with an equal volumetric ratio of 1:1:1. To assess the electrochemical performance, including cycling and rate capabilities, we utilized a LAND CT20001A battery tester. Finally, cyclic voltammetry (CV) and electrochemical impedance spectroscopy (EIS) measurements were conducted using an electrochemical workstation, the CHI660D.

## 3. Results and Discussion

### 3.1. Structure of Bi@C and Biomass Carbon

**Figure 2:**
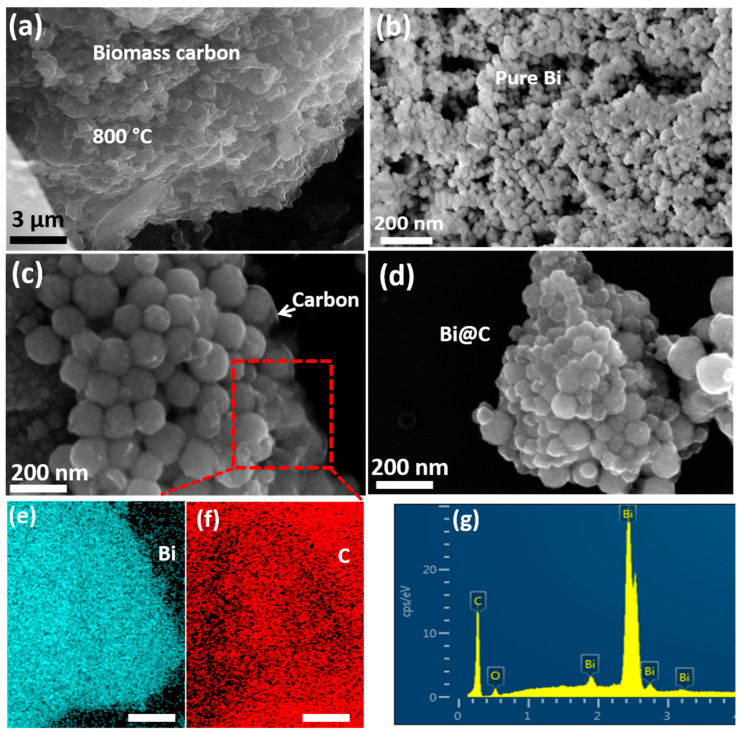
(**a**) SEM images of biomass carbon, (**b**) SEM image of pure Bi particles, (**c**) selected area for elemental mapping, (**d**) SEM image of composite Bi@C sample with different resolutions, (**e**,**f**) elemental mapping of Bi and carbon element, and (**g**) EDX of Bi@C composition.

In the composite Bi@C synthetization process, as illustrated in Figure 1, carbon is derived from a natural source and treated with Bi nanoparticles. The biomass-derived carbon was derived from sunflower seed husks. Potassium hydroxide (KOH) is a strong base commonly used as a chemical reagent. The KOH reagents were used to activate the carbon at a temperature of 800 °C. Additionally, the simple wet-chemical method was adopted to prepare the Bi@C nanocomposite, also shown in Figure S1. Following the calcination process, we verified the morphology and structure of the Bi@C material by using SEM characterization, as depicted in Figure 2. The particles are almost the same in size and have no difference, as shown in Figure 2b,c. Figure 2b shows the particles of the pure Bi product without treatment with biomass carbon coating. The ultrathin carbon layers played an important role in preventing the Bi particles from expanding in volume while lithium ions were inserted during cycling. Further, the biomass-derived carbon SEM image is exhibited in Figure 2a and Figure S2A. Based on Figure 2c, the desired elements Bi and C are homogeneous and evenly distributed in all the particles in the Bi@C composite, which indicates a good framework between particles and carbon. Further, the average particle size of Bi@C particles is 60–80 nm, as shown in Figure S2B. For pure Bi nanoparticles morphology, as shown in Figure 2d, the particles before coating are visible; there are no outer layers of carbon. In LIB anodes, it is established that small particles and a free space structure could deliver high electrochemical performance [28]. Based on the elemental mapping and EDX pattern in Figure 2e–g, the desired elements are present, and the particle distribution is consistent with the map. A unique morphology of the composition of Bi@C could improve the electrochemical performance of LIB anodes.

**Figure 3:**
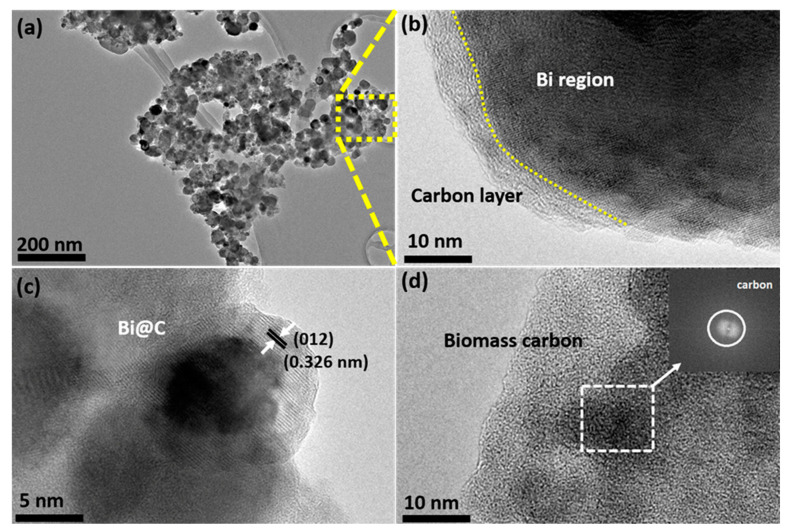
(**a**) TEM image of Bi@C, selected area; (**b**) two regions of Bi and carbon layer; (**c**) crystallinity of Bi@C sample; (**d**) TEM image of biomass-derived carbon and live FFT pattern of carbon.

For surface investigation, we employed high-resolution transmission electron microscopy (HRTEM) for TEM analysis. In Figure 3a, it has been shown in the high-resolution image that the particles are tightly arranged, indicating good structural stability even after 30 min of ultrasonication. In Figure 3b, we observe two visible and clearly defined regions: one exhibiting a crystalline path and the other comprising a carbon wall. The carbon wall is responsible for the protection of the Bi nanoparticles. Figure 3c presents the crystallinity, evidenced by the interlayer distance of 0.326 nm, which belongs to the (012) plane in the XRD pattern. Additionally, Figure 3d showcases the characteristic amorphous morphology of biomass-derived carbon, a confirmation further supported by TEM live FFT analysis. For pristine biomass-derived carbon, TEM images showed the typical carbon seen in Figure S3A–D.

### 3.2. Physical Structure Characterization

**Figure 4:**
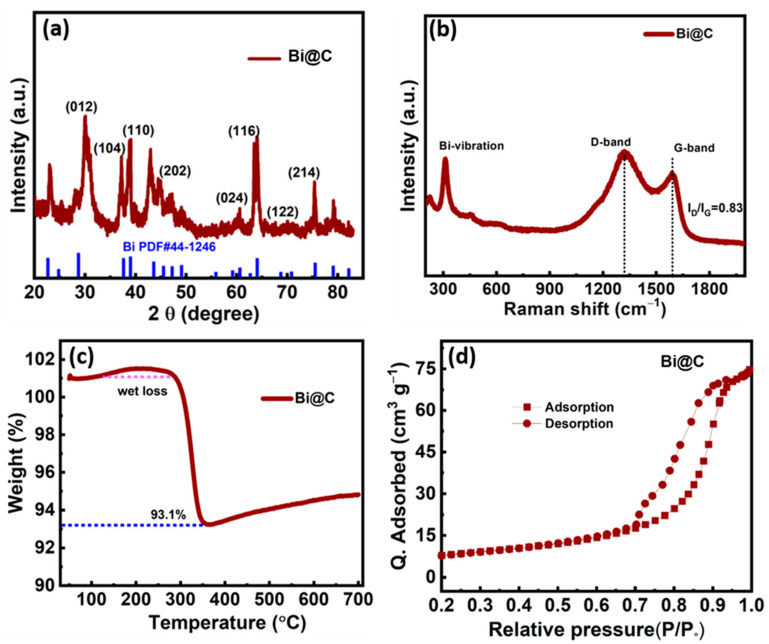
(**a**) XRD patterns of Bi@C composite, (**b**) Raman curves of Bi@C sample, (**c**) TGA of sample Bi@C, and (**d**) BET of Bi@C sample curves N_2_ sorption isotherms.

X-ray diffraction (XRD) patterns revealed the crystalline structures of the Bi@C samples, as shown in Figure 4a. The broad diffraction peaks at 2θ angles of 26.01, 37.86, 39.59, 48.71, 56.03, 61.99, 64.49, and 70.61° correspond to the (012), (104), (110), (202), (024), (116), (122), and (214) planes of the metal Bi phase, respectively. No additional peaks were observed alongside these main peaks, indicating the high material purity of the Bi@C samples. For typical biomass-derived carbon, the XRD pattern is demonstrated in Figure S4A, with the obvious peak of carbon at 24.7° belonging to the (002) plane. Regarding the Raman spectra (Figure 4b), the peaks observed at about 1363 and 1593 cm^−1^ represent the D and G bands, respectively. In order to determine the crystallinity of the material, the integration area of D peaks was compared with the integration area of G peaks; and the carbon pattern is also shown in Figure S4B. A reduced ID/IG value indicates a higher degree of graphitization [29]. In addition, the thermogravimetric analysis curves (TGA) were used to estimate the Bi element content in the Bi@C composites (Figure 4c). The increment in the carbonization temperature led to a significant increase in the Bi content, which was attributed to the agglomeration of bismuth metal. Even after removing moisture or water through wet processing, the carbon content remains at approximately 7% of the composition. As a result of the small amount of carbon coating encouraging the ultrathin layer buffering the structure, it might deliver excellent stability for anode LIBs. Figure 4d illustrates the isotherm of N_2_ adsorption and desorption on the prepared material to determine its surface area. According to the BET analysis, Bi@C has a surface area of 71.2 m^2^ g^−1^. Bi@C mesopores (with low P/P_0_) and macropores (with high P/P_0_) were well-developed according to N_2_ adsorption isotherms. The larger surface area provides higher energy density, faster charging and discharging, improved cycling stability, and better battery performance overall.

**Figure 5:**
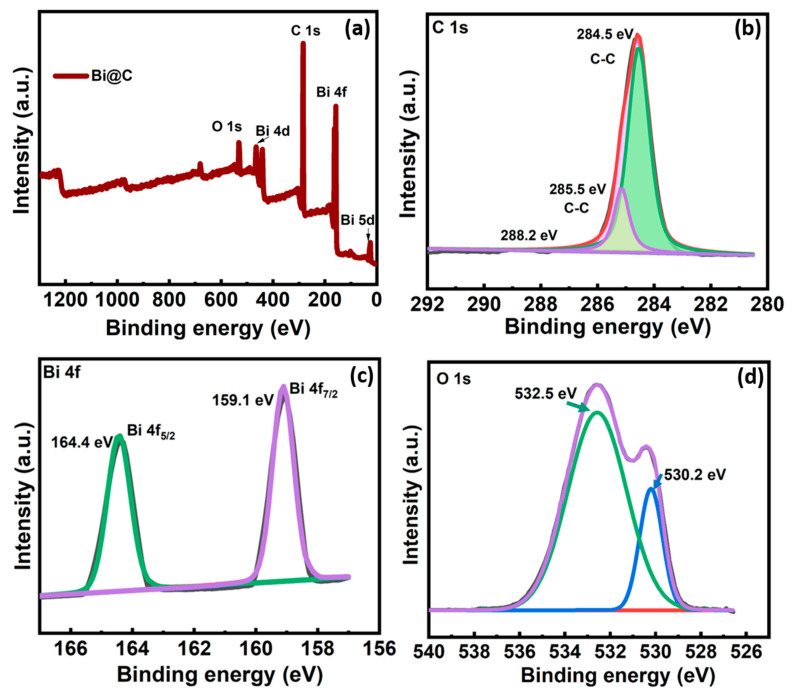
(**a**) XPS general spectrum of Bi@C composite of (**b**) Bi 4f, and (**c**) XPS spectra of C 1s. (**d**) XPS spectra of O 1s.

The X-ray photoelectron spectroscopy (XPS) analysis was carried out for a glimpse into the chemical bonding state of the Bi@C nanocomposite surface. In Figure 5a, the survey spectrum covers elements such as C, O, and Bi within the 0–1350 eV range. The deconvolution of the C1s spectrum in Figure 5b reveals three distinct peaks. The main peak at 284.5 eV is associated with sp^2^ hybridized graphitic carbon, whereas the additional two peaks at higher binding energies, figured 285.5 eV and 288.2 eV, correspond to C−O and O−C=O bonds [30]. Further examination with high-resolution spectra reveals Bi species characterized by two distinct peaks centered at 164.4 eV and 159.1 eV, corresponding to Bi 4f_5/2_ and Bi 4f_7/2_, respectively (Figure 5c) [31,32]. These features imply the predominant presence of oxygen and carbon on the carbon surface, resulting from the creation of oxygen-containing functional groups, including carboxyl (as depicted in Figure 5d) [33]. XPS peaks for O 1s 530.2 eV and 532.5 eV correspond to O-Bi and C-O-Bi, respectively. The robust binding of these functional groups to the carbon matrix could potentially serve as an anchor for the Bi particles. This porous carbon layer serves a dual purpose, enhancing both electron transfer and reducing the volume expansion of Bi nanoparticles during electro-adsorption.

### 3.3. Electrochemical Properties of Biomass Carbon, Pure Bi and Bi@C

**Figure 6:**
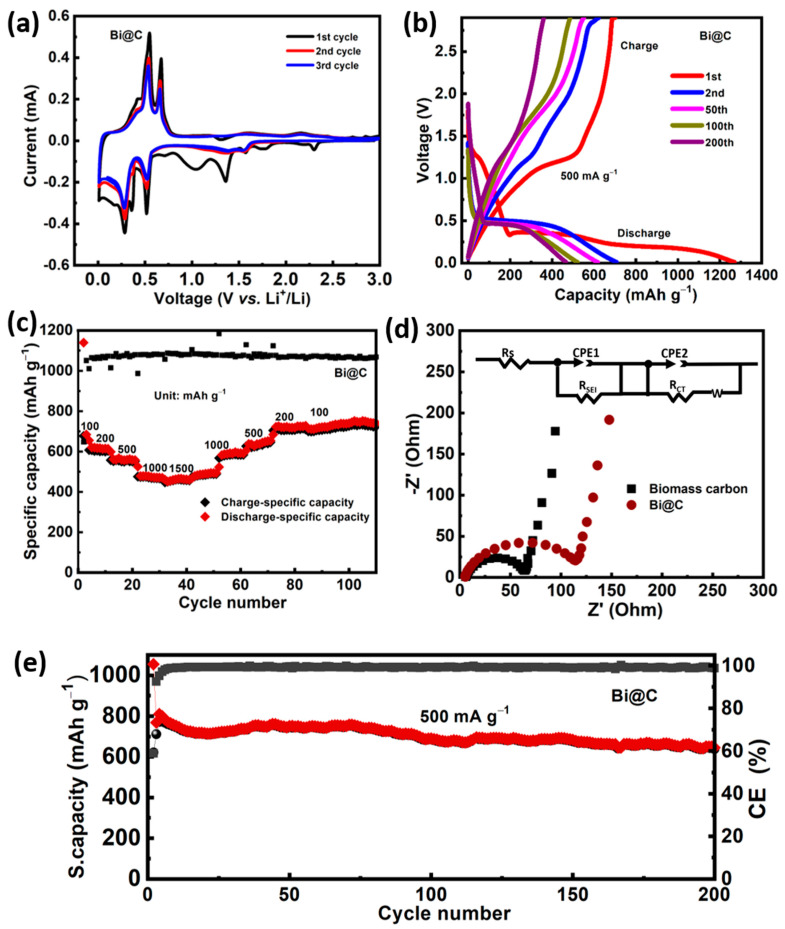
(**a**) the cyclic voltammetry patterns of the Bi@C nanocomposite at a scan rate of 0.1 mVs^−1^, (**b**) charge and discharge curves of Bi@C electrode at a current density of 500 mA g^−1^, (**c**) various current rates of the Bi@C electrode, (**d**) EIS Nyquist spectra of Bi@C composite and biomass-derived carbon, (**e**) cycling performance of the Bi@C electrode at current rate of 500 mA g^−1^.

The Bi@C composite is used as an anode material in lithium-ion batteries in order to evaluate its lithium storage performance. Figure 6a shows a cycle voltammeter (CV) curve for the Bi@C electrode for the first three cycles at a scan rate of 0.1 mV s^−1^. A wide reduction peak is observed in the initial cathode scan at 1.45 V vs. Li^+^/Li, likely caused by electrolyte decomposition resulting in solid electrolyte interphase (SEI) formation when the electrolyte decomposes [34,35,36]. In subsequent cathodic scans, peaks centered at 0.76 V and 0.64 V can be attributed to the formation of LiBi and Li_3_Bi [37,38]. Anodic peaks ranging from 0.62 to 0.91 V are associated with the gradual delithiation of Li_x_Bi alloys. A high reversibility lithiation/delithiation process has been demonstrated using Bi@C nanocomposite electrodes. Furthermore, the redox peaks exhibit consistent intensity and positions after the first cycle, indicating a high rate of reversibility. Figure 6b presents representative galvanostatic charge/discharge curves recorded at a current intensity of 500 mA g^−1^. Notably, the first discharge and charge exhibit specific gravimetric capacities were 10 mAh g^−1^ and 771 mAh g^−1^, demonstrating the high capacity of the Bi@C electrode. The initial discharge capacity also creates a compressed discharge plateau associated with the electrolyte’s decomposition. During charge/discharge processes, the plateau is gradually reduced along with lithiation/delithiation reactions, suggesting a shift from a primarily faradaic to a capacitive charge storage mechanism. It has been observed that batteries with alloy-type anodes are prone to this phenomenon. As a result of repeated nucleation reactions, Bi@C composites tend to become amorphous, resulting in a gradual decrease in lattice energy during both charge and discharge. Furthermore, volume alterations can also stimulate pseudocapacitive and capacitive reactions, which lead to a reduced size and an increased surface area. A wide range of current densities of 100 to 1500 mA g^−1^ are investigated in order to determine the rate performance of Bi@C electrodes (Figure 6c). Composite Bi@C electrodes have a much higher specific capacity at current densities of 100, 200, 500, 1000, and 1500 mA g^−1^, which deliver 701, 625, 560, 470, and 435 mAh g^−1^, respectively; for comparison, the cycling performance of pristine carbon electrode showed a stable performance of about 320 mAh g^−1^ for 250 cycles, as can be seen in Figure S5C. In this experiment, while current intensity was returned to 100 and 200 mA g^−1^, the specific capacity of the Bi@C electrode showed recovery up to levels exceeding 720 mAh g^−1^, indicating the impressive lithium storage kinetics and reversibility of Bi@C. A high current density, however, rapidly reduces the capacity of pure Bi particles, as shown in Figure S5A. This impressive lithium storage performance is due to the ultrathin coating nanocomposite structure of the Bi@C electrode. In addition, carbon in contact with the electrolyte may gradually develop a solid electrolyte interphase (SEI) when repeated charge and discharge cycles occur without significant volume expansion. Alternatively, the dynamic Bi core enclosed within the internal cavity can expand or contract, enhancing kinetics reactions in scenarios where the Bi core fragments and the active components are protected within the enclosed ultrathin layers, ensuring electrical conductivity.

The cycling performance of pure Bi and Bi@C electrodes is subsequently assessed using electrochemical impedance spectroscopy (EIS). As depicted in Figure 6d, both EIS spectra display a condensed semicircular feature in the high-medium frequency range, signifying the interfacial charge transfer resistance. Additionally, a diagonal line appears at lower frequencies, indicative of the customary Warburg behavior associated with the diffusion of Li^+^ ions. Figure 6d shows an equivalent circuit that can be used to model the spectra shown in the figure window. Carbon samples have a slightly smaller solution resistance than Bi@C. This is due to the nature of carbon, which has a smaller resistance than Bi@C. In addition to the R_S_ and R_SEI_, Bi@C electrodes have been calculated to have a higher R_ct_ value than pure biomass carbon derived from sunflower seed anodes. The pure Bi sample experiences a notable surge in interfacial impedance after 200 cycles. This can be attributed to the development of unstable solid electrolyte interphase (SEI) films and substantial volume fluctuations throughout the cycling process, aligning with the observed significant capacity deterioration. Table 1 shows the biomass carbon and Bi@C composite electrode, which shows the comparison values of a smaller charge transfer resistance of 67.31 Ω and 116.21 Ω, respectively. In the Bi@C electrode, a carbon layer covering the Bi nanoparticles may have contributed to favorable lithium-ion transportation. The excellent framework provides free channels for electron flow during LIB cycling.

Bi@C nanocomposite electrodes exhibit good cyclic stability under a 500 mA g^−1^ current density, except for the initial few cycles. It is believed that the initial instability of the cyclic curves results from the solid electrolyte interphase (SEI) layer forming and the electrode structure being optimized [39]. After 200 cycles, the reversible capacity reaches an impressive 730 mAh g^−1^, showcasing an outstanding capacity retention of 80% even after the 10th cycle. This performance stands in stark contrast to the pure Bi electrode, which retains only 26 mA h g^−1^ after 180 cycles, as illustrated in Figure 6e. The average loaded mass of a Bi@C electrode, determined by considering the mass of all electrodes on the copper current collector, amounts to 0.98 mg cm^−2^. The Bi@C composite’s cycling performance at 500 mA g^−1^ is shown in Figure 6e. When tested for 200 cycles at 500 mA g^−1^, the Bi@C nanocomposite maintains a capacity of 730.8 mAh g^−1^. Furthermore, Figure S5 illustrates the cyclic performance of the pure carbon matrix, demonstrating that the carbon matrix enhances the cycling stability in Bi@C electrodes. A comparison of the electrochemical properties of the Bi@C nanocomposite with those of Bi and biomass-derived carbon electrodes (as shown in Figure S5A,C) further highlights its structural benefits. In addition, Figure S5 shows the electrochemical performance of pristine carbon and pure Bi nanoparticles for comparison, clearly showing better results indicating the volume expansion problem with pure Bi electrodes during cycling. After ten cycles, cycling performance was significantly reduced and deteriorated. The coating of biomass-derived carbon played an important role in cycling stability, making Bi@C a good candidate for anode LIBs.

**Figure 7:**
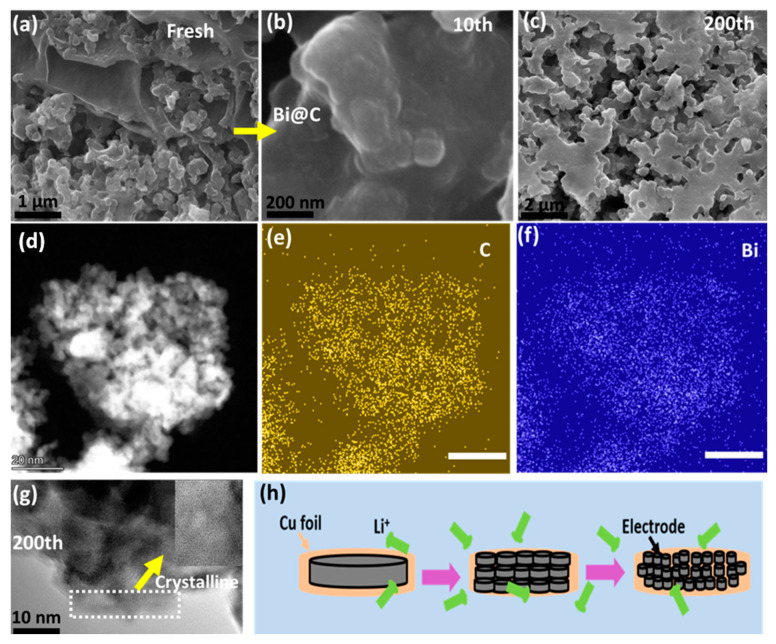
(**a**) After cycling SEM image of Bi@C composite of fresh sample; (**b**) SEM image of the sample after 10 cycles; (**c**) structure stability after 200 cycles; (**d**) HAADF image of the Bi@C sample; (**e**) elemental mapping of C, (**f**) Bi element; (**g**) structure crystallinity path after 200 cycles; and (**h**) schematic diagram of lithium-ion insertion during cycling.

The unique structure of the Bi@C composite is undoubtedly responsible for its outstanding performance. Figure 7 illustrates the morphological and structural changes in the Bi@C composite electrodes following different cycles. After the first cycle, a preliminary solid electrolyte interphase (SEI) layer is evident on the Bi@C composite (Figure 7a,b). Over 100 cycles, this layer gradually transforms into smaller particles (Figure 7c,d), eventually stabilizing into a coherent network structure after 200 cycles (Figure 7e). The phase images provide additional insights, with the initial electrode displaying extensive phase separation that diminishes in size and becomes more uniform after 200 cycles. This observation aligns with developing a thicker SEI layer on the Bi@C composite anodes and corroborates the morphological evolution observed in SEM and TEM images. For deep investigation, HAADF analysis illustrated that Bi@C nanoparticles have a regular, homogeneous, and equally distributed distribution (Figure 7d). Additionally, Figure 7e,f show the desired elements C and Bi. Similarly, elemental mapping images indicate that Bi remains in carbon networks after cycling. Bi@C electrodes exhibited crystallinity path stability after 200 cycles, as shown in Figure 7g. In order to maintain consistent cycling performance, this strong network structure between carbon and Bi nanoparticles has mitigated strain and minimized volume changes during charging and discharging [40,41,42]. Lithium insertion in the electrode for free ion transport is shown in Figure 7h. The structure is stable before and after cycling, indicating that volume changes can be restrained and excellent electrochemical performance can be achieved.

## 4. Conclusions

In this study, we successfully derived biomass carbon from sunflower seed husks, and then carbon was coated onto bismuth nanoparticles via a simple chemical method. For this proposed electrode, Bi nanoparticles are uniformly distributed within carbon networks due to their simple growth mechanism, compact coating, and free space structure; as a result, the composite Bi@C offered a stable performance. The Bi@C composite emerged as a standout choice for an anode material, showcasing excellent durability with an extended cycle life of 200 cycles at a high current density of 500 mA g^−1^, delivering a high capacity of 731.8 mAh g^−1^. Moreover, under the challenging conditions of a high current rate of 1500 mA g^−1^, the Bi@C composite anode continued to exhibit a high performance of 435 mAh g^−1^. This suggests that our fabricated structure is stable, even after extensive cycling, which underscores the effectiveness of the naturally compact carbon layers in mitigating volume changes. For the future, the simple synthetization method and robust framework, the Bi and biomass carbon (Bi@C) composite, holds substantial promise and could be used for practical large-scale applications.

## Figures and Tables

**Table 1:** The impedance parameters.

Sample	R_s_ (Ω)	R_ct_ (Ω)
Biomass-derived carbon	2.612	67.31
Bi@C	2.976	116.21

## Data Availability

Not applicable.

## Supplementary Materials

The following supporting information can be downloaded at: https://www.mdpi.com/article/10.3390/ma16206628/s1. Figure S1. Schematic diagram of biomass-derived carbon and Bi@C composite. Figure S2. (A) SEM image of biomass-derived carbon; (B) the average size of particles of Bi@C composite. Figure S3. (A) TEM image of biomass-derived carbon, and (B–D) different resolutions of carbon nanoparticles. Figure S4. (A) XRD spectra of biomass-derived carbon and (A,B) RAMAN spectra of biomass-derived carbon. Figure S5. (A) Cycling performance of pure Bi nanoparticles for 200 cycles, (B) different current rate performances of biomass-derived carbon, and (C) cycling performance at a current rate of 500 mA g^−1^ of biomass-carbon sample for 250 cycles.
