# Supplementary material for: Bismuth-Nanoparticles-Embedded Porous Carbon Derived from Seed Husks as High-Performance for Anode Energy Electrode
Source: Materials (Basel). 2023 Oct 10;16(20):6628. doi: 10.3390/ma16206628 (PMC10608430; doi:10.3390/ma16206628)
Supplement: Supplementary file 1 [file materials-16-06628-s001.zip › materials-2652097-SI.pdf]

Supplementary

Bismuth-Nanoparticles-Embedded Porous Carbon Derived from Seed Husks as High-Performance for Anode Energy Electrode

Wasif ur Rehman [1], Umar Farooq [2,*], Muhammad Zain Yousaf [3] and Ali Altalbe [4,5]

[1] Hubei Key Laboratory of Energy Storage and Power Battery, School of Mathematics, Physics and Opto-Electronic Engineering, Hubei University of Automotive Technology, Shiyan 442002, China; wasif@huat.edu.cn
[2] Key Laboratory of the Ministry of Education for Advanced Catalysis Materials, Zhejiang Normal University, Jinhua 321017, China
[3] School of Electrical and Information Engineering, Hubei University of Automotive Technology, Shiyan 442002, China; mzainy1@gmail.com
[4] Department of Computer Science, Prince Sattam Bin Abdulaziz University, Al-Kharj 11942, Saudi Arabia; a.altalbe@psau.edu.sa
[5] Faculty of Computing and Information Technology, King Abdulaziz University, Jeddah 21589, Saudi Arabia
* Correspondence: umaricup@zjnu.edu.cn

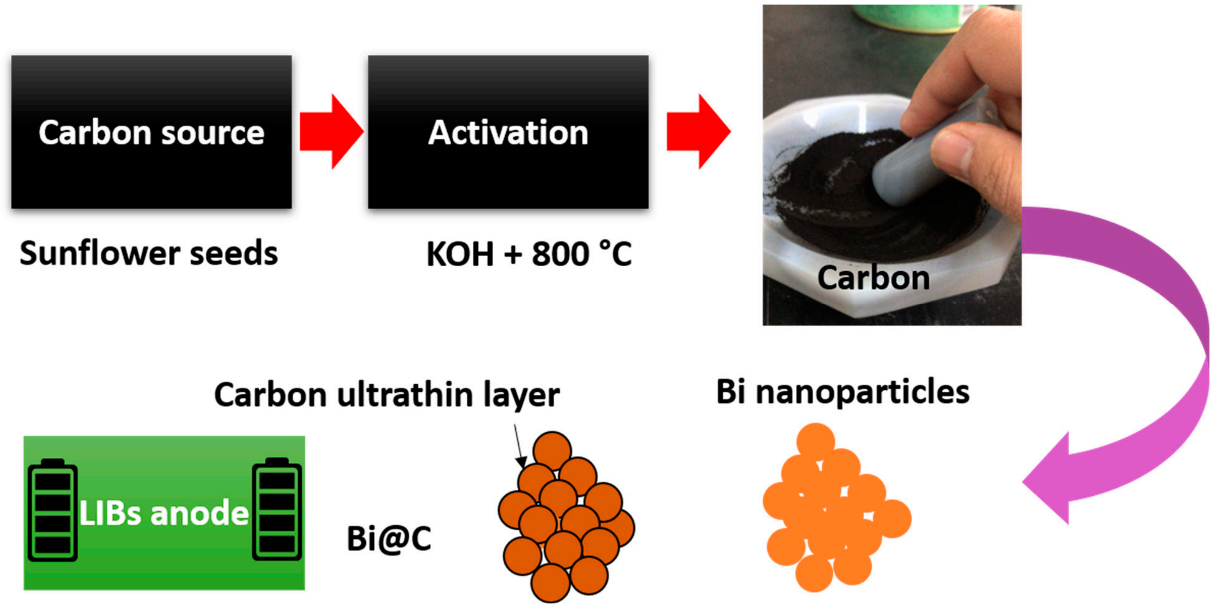

Figure S1. Schematic diagram of Biomass-derived carbon and Bi@C composite.

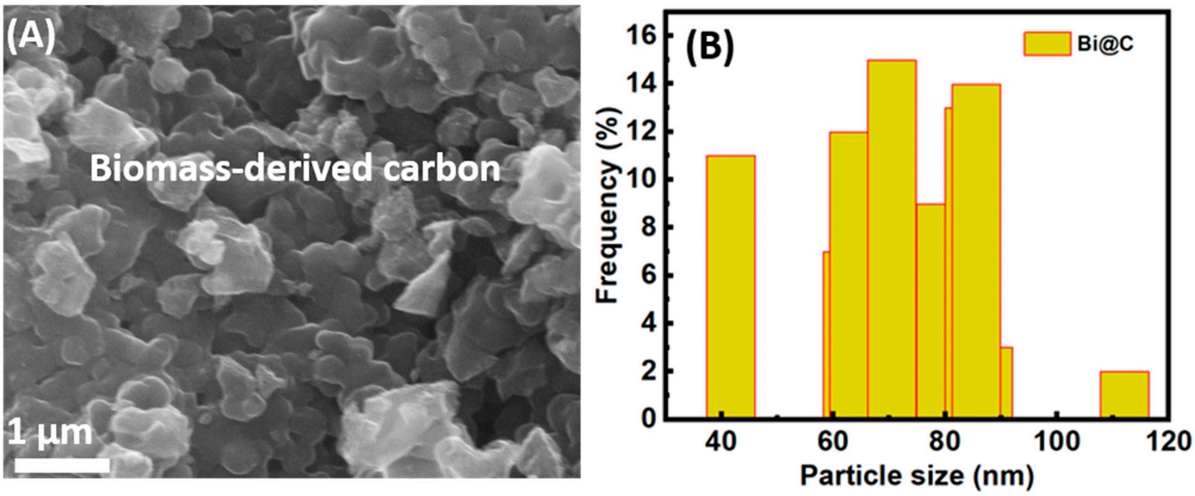

Figure S2. (A) SEM image of Biomass-derived carbon (B) the average size of particles Bi@C composite.

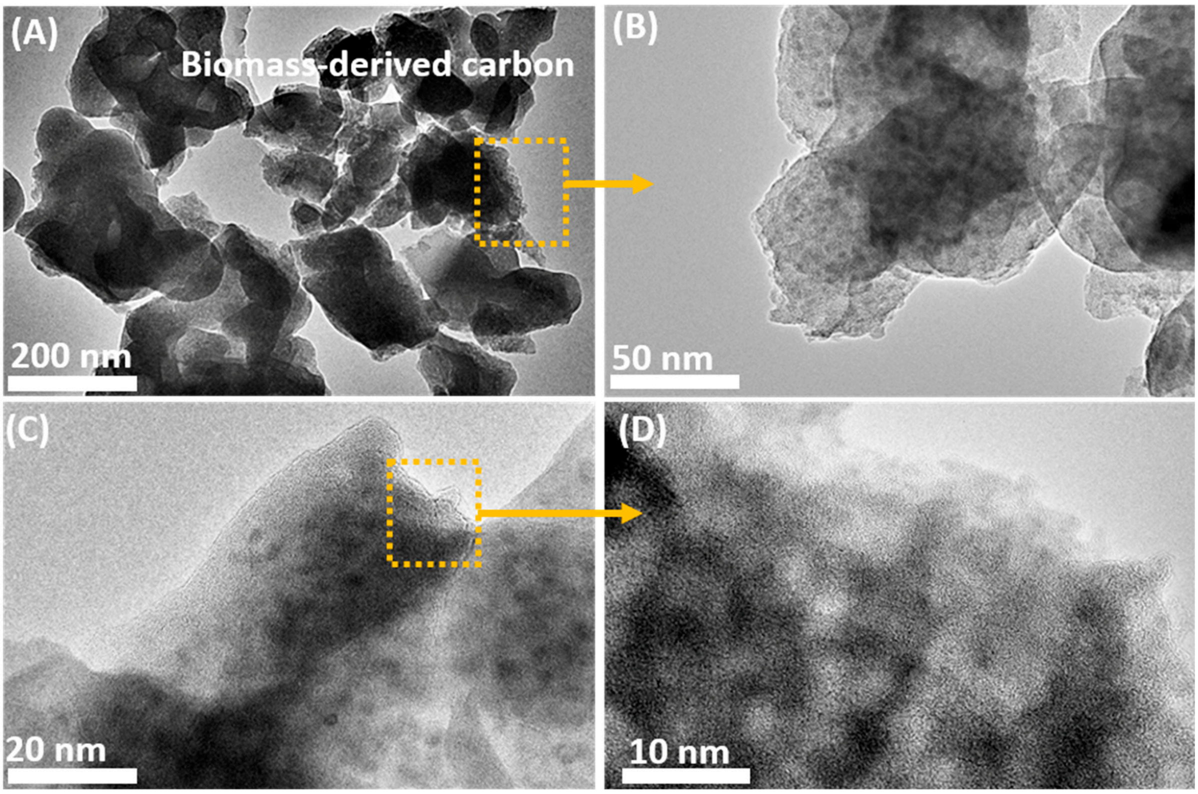

Figure S3. (A) TEM image of Biomass-derived carbon and (B–D) different resolution of carbon nano particles.

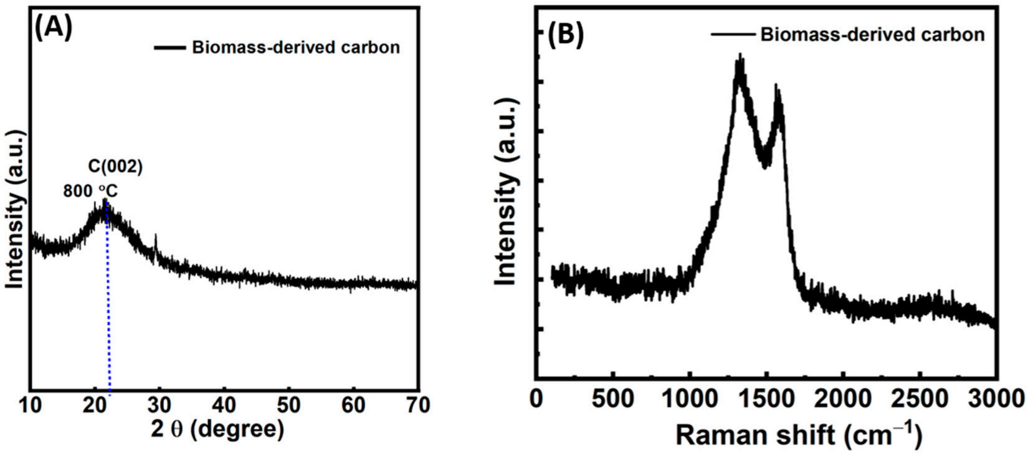

Figure S4. (A) XRD spectra of Biomass-derived carbon and (A,B) RAMAN spectra of Biomass-derived carbon.

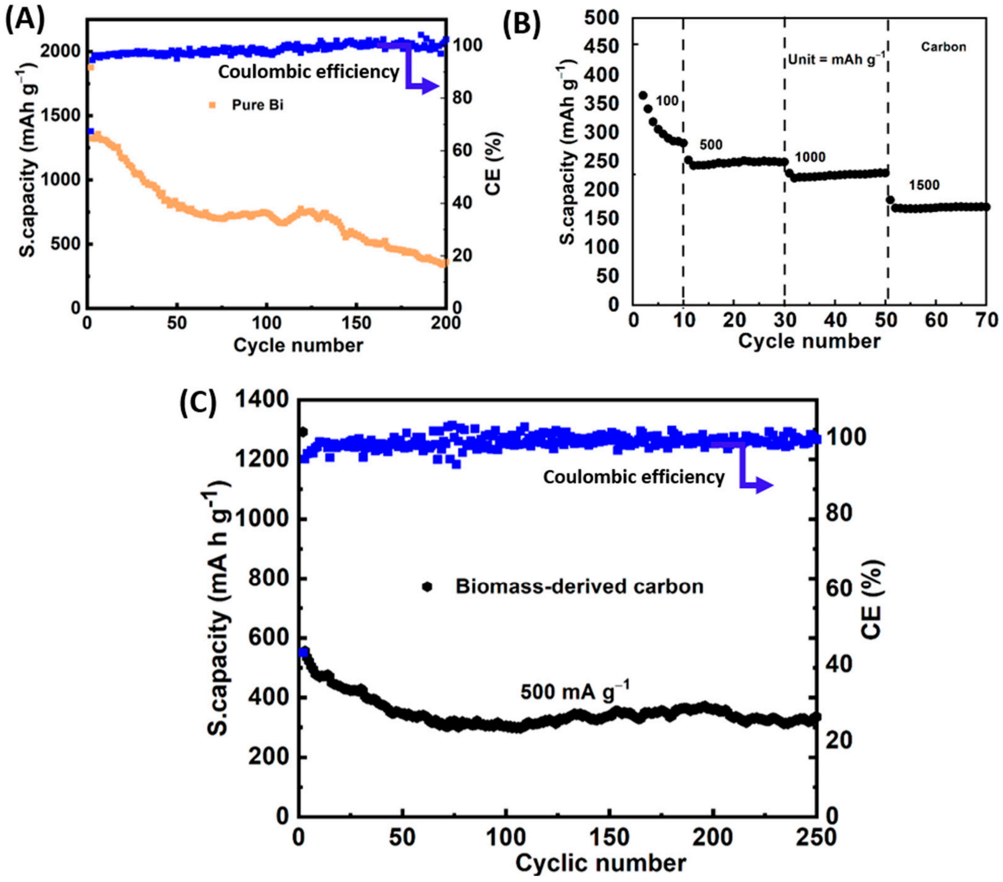

Figure S5. (A) cycling performance of pure Bi nanoparticles for 200 cycles, (B) different current rate performance of Biomass-derived carbon and (C) cycling performance at a current rate of 500 mA g^{-1} of biomass-carbon sample for 250 cycles.
